# Live nontypeable *Haemophilus influenzae* exacerbates respiratory syncytial virus-associated lower airway inflammation

**DOI:** 10.1186/s12931-026-03774-4

**Published:** 2026-06-20

**Authors:** Eigo Kawahara, Kohei Morimoto, Md. Shafiqul Islam, Hiroshi Yamada, Takafumi Sekiguchi, Mika Morita, Junichi Mitsuyama, Yoshitomo Morinaga

**Affiliations:** 1 Center for Advanced Antibody Drug Development, University of Toyama, 2630 Sugitani, Toyama, 930-0194 Japan; 2 Department of Microbiology, Faculty of Medicine, Academic Assembly, University of Toyama, 2630 Sugitani, Toyama, 930-0194 Japan; 3 Department of Laboratory Medicine, Toyama University Hospital, 2630 Sugitani, Toyama, 930-0194 Japan; 4 Present Address: Division of Molecular Target and Gene Therapy Products, National Institute of Health Sciences, 3-25-26 Tonomachi, Kawasaki-ku, Kawasaki, Kanagawa 210-9501 Japan

**Keywords:** Respiratory syncytial virus (RSV), Nontypeable *Haemophilus influenzae* (NTHi), Lower respiratory tract diseases, CD8^+^ T cells, IL-6

## Abstract

**Background:**

Respiratory syncytial virus (RSV) is a major cause of lower respiratory tract disease in infants and immunocompromised adults, and secondary bacterial infections are recognized as important contributors to disease severity. Recent microbiome studies have shown that *Haemophilus influenzae* is frequently co-detected in severe RSV cases and ranks among the bacteria most closely correlated with disease severity; however, its causal role and underlying mechanisms remain unclear. Here, we used a murine model to examine the manner in which *H. influenzae* influences RSV-associated airway inflammation.

**Methods:**

We established a murine respiratory infection model by infecting mice with RSV and subsequently intratracheally inoculating them with live, ultraviolet (UV)-killed, or heat-killed nontypeable *H. influenzae* (NTHi). Body weight, viral and bacterial loads, bronchoalveolar lavage fluid (BALF) cytokine levels, and immune cell populations were analyzed.

**Results:**

Live NTHi inoculation following RSV infection induced severe lower airway inflammation, characterized by pronounced body weight loss and lung injury along with increased CD8^+^ T-cell accumulation and enhanced interleukin (IL)-6 inflammatory mediator production in BALF. Additionally, CD8^+^ T-cell depletion modestly attenuated body weight loss, altered the recruited innate immune cell composition, and reduced BALF IL-6 levels. However, RSV-infected mice inoculated with UV- or heat-killed NTHi exhibited CD8^+^ T-cell accumulation without developing body weight loss, which suggested that CD8^+^ T-cell accumulation alone may be insufficient to drive full disease manifestation. In contrast to that observed for non-viable NTHi, live NTHi induced robust neutrophil recruitment along with strong production of inflammatory mediators. Additionally, BALF IL-6 levels showed a strong negative correlation with body weight; hence, IL-6 was identified as a potential marker associated with disease severity.

**Conclusions:**

This mouse model shows that pulmonary inoculation of live NTHi after RSV infection induces severe lower airway inflammation partially involving CD8^+^ T cells and that IL-6 is a potential biomarker associated with disease severity. Overall, this study suggests a novel pathological role for NTHi in the exacerbation mechanism of secondary bacterial infection in RSV infection.

**Supplementary Information:**

The online version contains supplementary material available at https://doi.org/10.1186/s12931-026-03774-4.

## Background

Respiratory syncytial virus (RSV) infects almost all infants by 2 years of age, and lifelong reinfections occur frequently [1, 2]. RSV typically infects the upper respiratory tract and causes mild cold-like symptoms such as rhinorrhea and coughing [1, 3]. However, in infants and immunocompromised adults, the infection may spread to the lower respiratory tract and cause severe respiratory illnesses such as pneumonia and bronchiolitis [1, 4]. Globally, RSV is responsible for millions of hospitalizations and a substantial number of deaths annually, which impose a significant public health burden [1, 5]. Although recent advances in RSV vaccines and monoclonal antibodies have led to several clinical approvals [6, 7], no definitive antiviral therapy has been established, and treatment for severe disease remains largely supportive. Thus, elucidating the mechanisms underlying RSV disease severity and developing new therapeutic strategies remain urgent priorities.

Secondary bacterial infections following RSV infection are recognized as important contributors to disease severity [3, 4, 8]. As culture techniques preferentially detect bacteria that grow readily under laboratory conditions, they fail to capture the full spectrum of microbial communities inhabiting the respiratory tract. With the increased application of next-generation sequencing (NGS), numerous studies analyzing the respiratory microbiota of patients with severe RSV infection have reported frequent detection of *Haemophilus influenzae* [9–12]. *H. influenzae* is a Gram-negative coccobacillus that is primarily identified as a respiratory tract pathogen [13, 14]. Encapsulated strains are associated with invasive bacterial infections. Particularly, type b strains have shown a pronounced declined in prevalence in several countries following the introduction of the *H. influenzae* type b conjugate vaccine [13–15]. However, this vaccine does not elicit any protective effects against non-typeable *H. influenzae* (NTHi)-caused infections, which have been showing increase in prevalence [13–15]. NTHi is a major pathogen responsible for otitis media, chronic respiratory infections, and lower respiratory diseases [14, 16]. However, NTHi infection alone rarely causes severe disease. Whether the *H. influenzae* co-detected in patients with severe RSV infection is NTHi remains unclear; nevertheless, the high coverage of the Hib vaccine and predominance of NTHi among *H. influenzae* strains detected in respiratory infections in the countries to which the study populations belong suggest that NTHi likely accounts for the majority of these detections [13, 17]. Therefore, the co-occurrence of NTHi with viral infection may exacerbate respiratory pathology.

Primarily using in vitro systems, experimental studies have demonstrated interactions between RSV and NTHi. Exposure to NTHi following RSV infection results in significantly increased adhesion of live NTHi to airway epithelial cells [18–21]. NTHi has been shown to bind directly to the RSV G glycoprotein, and this finding provides a molecular basis for the enhanced bacterial attachment during viral infection [22]. Furthermore, RSV infection promotes the upregulation of NTHi type IV pilus expression, particularly that of *pilA*, which further facilitates bacterial adherence and colonization [23]. These in vitro findings collectively indicate that RSV infection creates a permissive epithelial environment that promotes NTHi attachment and virulence-associated phenotypes. However, these studies are largely based on simplified in vitro models, which do not fully recapitulate the complex airway architecture, immune cell interactions, and dynamic host–pathogen relationships present in vivo. Additionally, in vivo experimental validation remains limited. Consequently, the extent to which in vitro observations reflect the pathological processes occurring in the lower respiratory tract during RSV-associated disease remains incompletely understood.

Despite both clinical and experimental evidence suggesting interactions between RSV and NTHi, whether their co-occurrence directly contributes to the development of severe lower respiratory tract inflammation remains unclear. To address this gap, we established a murine respiratory infection model by administering NTHi intratracheally after RSV infection. Using this model, we systematically analyzed pathogen loads, immune cell dynamics, inflammatory mediator production, and lung pathology to elucidate the mechanisms underlying severe RSV-associated airway inflammation.

## Methods

### RSV progression and enrichment

RSV strain A2 was propagated using HEp-2 cells, as previously reported [24, 25]. Briefly, for RSV propagation, one plaque forming unit (PFU) of RSV per 10^2^ HEp-2 cells was added to 70–80% confluent HEp-2 monolayers and incubated at 37 °C under 5% CO_2_ for 7 h with hourly shaking. Then, the culture medium was replaced with fresh medium. After incubation at 37 °C under 5% CO_2_ for 5 days, the cells were freeze–thawed to collect the virus, and the supernatant was collected by centrifuging twice at 700 × *g* for 5 min at 4 °C. For RSV enrichment, the supernatant was transferred to an ultracentrifuge tube, and 1 mL of phosphate-buffered saline (PBS) containing 20% sucrose was added to the bottom of the tube, followed by centrifugation at 71,000 × *g* for 3.5 h at 4 °C. The precipitated virus was resuspended in PBS, aliquoted, and stored at −80 °C for later use. The viral titers were measured using a plaque assay as previously reported [25]. Briefly, the viral diluent was added to 90% confluent HEp-2 monolayers and incubated at 37 °C under 5% CO_2_ for 2 h. After incubation, the culture medium was replaced with fresh medium containing 0.6% carboxymethylcellulose and incubated for a further 3 days at 37 °C under 5% CO_2_. Subsequently, the cells and viruses were fixed with methanol. The fixed viruses were treated with goat anti-RSV polyclonal antibody (catalog number: AB1128, dilution 1/500; Merck Millipore, Darmstadt, Germany) and horseradish peroxidase-conjugated donkey anti-goat IgG polyclonal antibody (catalog number: A15999, dilution 1/75; Thermo Fisher Scientific, Hampton, NH, USA). The plaques were visualized using 4-chloro-1-naphthol (Tokyo Chemical Industry, Tokyo, Japan) and counted.

### NTHi isolation and identification

*H. influenzae* strain UT-16,973 is a clinical isolate obtained from a patient at the Department of Clinical Laboratory, Toyama University Hospital. The bacterium was cultured overnight on chocolate agar plates (Kyokuto Pharmaceutical Industrial, Tokyo, Japan) at 37 °C under 5% CO_2_. After incubation, bacterial colonies were suspended in sterile saline containing 20% glycerol. Glycerol stocks were prepared and stored at −80 °C for later use. For DNA extraction, the bacterial cells were suspended in 50 µL of 0.05 M NaOH and heated at 95 °C for 15 min. The lysate was neutralized by adding 11 µL of 1 M Tris-HCl (pH 8.0). To confirm that the isolate was NTHi, the solution was diluted 10-fold with nuclease-free water and subjected to polymerase chain reaction (PCR) analysis using AmpliTaq Gold 360 Master Mix (Thermo Fisher Scientific). Previously reported primers were used, and these are listed in Supplementary Table S1 [26]. Genomic DNA from *H. influenzae* serotype b (ATCC 10211) was used as the positive control. The PCR cycling conditions were as follows: initial denaturation at 95 °C for 10 min; 35 cycles of denaturation at 95 °C for 30 s, annealing at 55 °C for 30 s, and extension at 72 °C for 60 s; and final extension at 72 °C for 7 min. Then, the reaction was held at 4 °C. The PCR products were analyzed by performing agarose gel electrophoresis using 2% Agarose S (NIPPON GENE, Tokyo, Japan) prepared in 0.5× Tris-borate-EDTA (TBE) buffer (Nacalai Tesque Inc., Kyoto, Japan). After electrophoresis, the gels were stained with an ethidium bromide solution for 30 min. DNA bands were visualized under UV illumination and imaged using the ChemiDoc Go Imaging System (Bio-Rad Laboratories, Hercules, CA, USA) (Supplementary Fig. S1).

### Preparation of live and killed NTHi

Glycerol stocks of NTHi were streaked onto chocolate agar plates and incubated at 37 °C under 5% CO_2_ for 20 h. Bacterial colonies were harvested and suspended in Brain Heart Infusion (BHI) broth (BD Biosciences, Sparks, MO) supplemented with 10 µg/mL hemin and 10 µg/mL β-nicotinamide adenine dinucleotide (β-NAD^+^, oxidized form). The bacterial suspension was adjusted to a 0.2 optical density at 600 nm (OD_600_) at a 10-mL final volume, as measured using a GloMax Discover Microplate Reader (Promega, Madison, WI, USA). The culture was incubated at 37 °C with shaking at 250 rpm for 4 h, after which the bacteria were collected by centrifugation at 3,000 × *g* for 4 min, followed by washing twice with sterile saline. The final pellet was resuspended in 10 mL of saline, and the bacterial concentration was adjusted to 2 × 10^7^ colony-forming units (CFU)/mL based on OD_600_ measurements, assuming that an OD_600_ of 0.8 corresponded to 5 × 10^9^ CFU/mL. To validate this estimation, the bacterial suspensions obtained after culturing were serially diluted, plated onto chocolate agar plates, and incubated at 37 °C under 5% CO_2_ to determine the viable CFU counts. For preparation of UV-killed NTHi, the bacterial suspensions were exposed to UV irradiation for 2 h [27]. For obtaining heat-killed NTHi, the bacterial suspensions were incubated at 56 °C for 30 min [28]. Complete bacterial killing was confirmed by plating on chocolate agar plates.

### Mouse respiratory infection model

Six-week-old female BALB/c mice were purchased from SLC (Hamamatsu, Shizuoka, Japan). They were housed with *ad libitum* access to food and water. Then, the mice were randomly assigned to the various experimental groups before infection or antibody treatment. On day −7, the mice were put under anesthesia with a mixture of midazolam (2.4 mg/kg) (Maruishi Pharmaceutical Co. Ltd., Osaka, Japan), butorphanol tartrate (3 mg/kg) (Meiji, Tokyo, Japan), and medetomidine hydrochloride (0.18 mg/kg) (ZENOAQ, Fukushima, Japan) and intranasally inoculated with 1.0 × 10^5^ PFU of RSV in 30 µL of PBS (15 µL to each nostril). Subsequently, the mice were awakened from anesthesia with atipamezole hydrochloride (0.18 mg/kg) (ZENOAQ). On day 0, the mice were intratracheally inoculated with 1.0 × 10^6^ CFU of live, UV-killed, or heat-killed NTHi in 50 µL of saline using an Angiocath Peripheral Venous Catheter (20 G; BD Biosciences) with a 1-mL syringe under visualization with an illuminated ear pick, as previously described [29]. The mice were anesthetized during this procedure and awakened by administering atipamezole hydrochloride after inoculation. After NTHi inoculation, the mice were anesthetized and euthanized. Bronchoalveolar lavage was performed, and the bronchoalveolar lavage fluid (BALF; total volume of 1 mL per mouse) and lung tissues were collected for subsequent analyses. To determine bacterial burden in the airways, the BALF samples were serially diluted and plated onto chocolate agar plates, which were incubated overnight at 37 °C in an atmosphere containing 5% CO_2_, and NTHi colonies were counted to quantify the CFUs.

### LDH assay

The BALF was centrifuged at 600 × *g* for 5 min, and lactate dehydrogenase (LDH) level in the supernatant was measured using the Cytotoxicity LDH Assay Kit-WST (Dojindo, Kumamoto, Japan) according to the manufacturer’s instructions. Absorbance was measured at OD_490_ using a GloMax Discover Microplate Reader.

### Histopathology analysis

The excised lungs were fixed using intratracheal instillation of 10% phosphate-buffered formalin until full lung inflation was achieved. Paraffin embedding, sectioning, and hematoxylin and eosin (H&E) staining were performed by Morphotechnology Co. Ltd. (Hokkaido, Japan). A pathology score was determined based on four parameters of pulmonary inflammation: peribronchiolitis (inflammatory cell infiltration around the bronchioles), perivasculitis (inflammatory cell infiltration around the small blood vessels), interstitial pneumonia (inflammatory cell infiltration and thickening of alveolar walls), and alveolitis (cells within the alveolar spaces). The slides were scored blindly on a severity scale of 0–4, as previously described [30].

### Measurement of RSV levels

The BALF was centrifuged at 600 × *g* for 5 min, and total RNA was extracted from the supernatant using the NIPPONGENE ISOSPIN RNA Virus (NIPPON GENE) according to the manufacturer’s instructions. Reverse transcription was performed using the ReverTra Ace qPCR RT Master Mix with gDNA Remover (TOYOBO, Osaka, Japan) to synthesize complementary DNA (cDNA). Real-time quantitative polymerase chain reaction (qPCR) was performed by amplifying the RSV nucleoprotein (RSV N) gene in a QuantStudio 6 Pro Real-Time PCR System (Thermo Fisher Scientific) using the THUNDERBIRD Next SYBR qPCR Mix (TOYOBO) and primers listed in Supplementary Table S1. The absolute RSV levels were calculated as PFU equivalents by generating a standard curve based on the serial dilutions of the cDNA synthesized from the RNA purified from the RSV stocks with known PFU titers.

### Detection of cytokines and chemokines

Cytokine and chemokine levels in the BALF, including those of interleukin (IL)-6, tumor necrosis factor (TNF)-α, IL-1β, IL-12p70, IL-10, interferon (IFN)-γ, IFN-β, IFN-α, granulocyte-macrophage colony-stimulating factor (GM-CSF), C–X–C motif chemokine ligand 1 (CXCL1), C–C motif chemokine ligand 2 (CCL2), CCL5, and CXCL10, were measured using the LEGENDplex Mouse Anti-Virus Response Panel (13-plex) with a V-bottom plate (BioLegend, San Diego, CA, USA) according to the manufacturer’s instructions. The data were acquired using a FACSCelesta flow cytometer (BD Biosciences).

### Flow cytometry

For longitudinal analysis of immune cells in the BALF, the collected BALF samples were centrifuged at 600 × *g* for 5 min, and the cell pellets were washed with staining buffer comprising PBS containing 2% fetal bovine serum (FBS), 1 mM EDTA, and 0.05% sodium azide. Subsequently, the cells were incubated in the dark for 15 min at 4 °C in a staining buffer containing Fixable Viability Dye eFluor 780 (catalog number: 65-0865-18, dilution 1:1000; Thermo Fisher Scientific) and fluorochrome-conjugated antibodies listed in Supplementary Table S2. The absolute cell numbers were calculated using Precision Count Beads (BioLegend), which were added immediately before flow cytometric acquisition performed using an FACSCelesta flow cytometer. The data were analyzed without blinding to the experimental groups using FlowJo software version 10.10 (FlowJo LLC, Ashland, OR, USA). For phenotypic analysis of CD8^+^ T cells, brefeldin A (Tokyo Chemical Industry) was prepared as a 10 mg/mL stock solution in dimethyl sulfoxide (DMSO) and diluted with PBS to a final injection volume of 500 µL per mouse. The mice were administered brefeldin A intraperitoneally at a dose of 250 µg per mouse 6 h prior to dissection on day 2 after NTHi inoculation to inhibit cytokine secretion. Then, they were anesthetized and euthanized, and the BALF was collected and centrifuged at 600 × *g* for 5 min. The cell pellets were washed with staining buffer. The surface antigens were stained by incubating the cells in the dark for 15 min at 4 °C in a staining buffer containing Fixable Viability Dye eFluor 780 and fluorochrome-conjugated antibodies listed in Supplementary Table S2. Next, intracellular cytokine staining was performed using the BD Cytofix/Cytoperm Fixation/Permeabilization Solution Kit (BD Biosciences) according to the manufacturer’s instructions. The cells were permeabilized and incubated with fluorochrome-conjugated antibodies listed in Supplementary Table S2. The absolute cell numbers were calculated using Precision Count Beads, which were added immediately before flow cytometric acquisition performed using an FACSCelesta flow cytometer. The data were analyzed without blinding to the experimental groups using FlowJo software version 10.10.

### Depletion of CD8^+^ T cells

Mice were intranasally inoculated with RSV on day −7. On day −1, the mice were injected intraperitoneally with 200 µg of rat anti-mouse CD8β antibody (clone: Lyt 3.2, catalog number: A2137; Selleck Chemicals, Houston, TX, USA) or rat IgG1 isotype control (clone: HRPN, catalog number: A2119; Selleck Chemicals) in 200 µL PBS. On day 0, the mice were intratracheally inoculated with live NTHi. On day 2 post NTHi inoculation, the mice were anesthetized and euthanized, and the BALF and lung tissues were collected for subsequent analyses.

### Statistical analysis

Statistical analyses were performed using the GraphPad Prism 10 software (GraphPad Software, San Diego, CA, USA). No statistical methods were used to predetermine sample size. Rather, they were determined based on previous studies that used similar respiratory infection models, preliminary observations, feasibility considerations, and ethical efforts to minimize animal use. The data are expressed as the means ± standard deviation (SD) or as medians. Comparisons between two groups were performed using an unpaired two-tailed Welch’s t test. For comparisons involving multiple groups and two independent variables, two-way analysis of variance (ANOVA) followed by Sidak’s or Dunnett’s multiple comparison test was applied, as appropriate. For comparisons among multiple groups with a single independent variable, one-way ANOVA followed by Tukey’s multiple comparison test was used. Correlations between variables were assessed using Spearman’s rank correlation test. Statistical significance was defined as *P* < 0.05. Detailed statistical information including sample sizes, exclusion criteria, statistical tests, assumptions, and number of independent experiments for each experiment is provided in the corresponding figure legends.

## Results

### NTHi inoculation following RSV infection induces severe lower airway inflammation

**Fig. 1:**
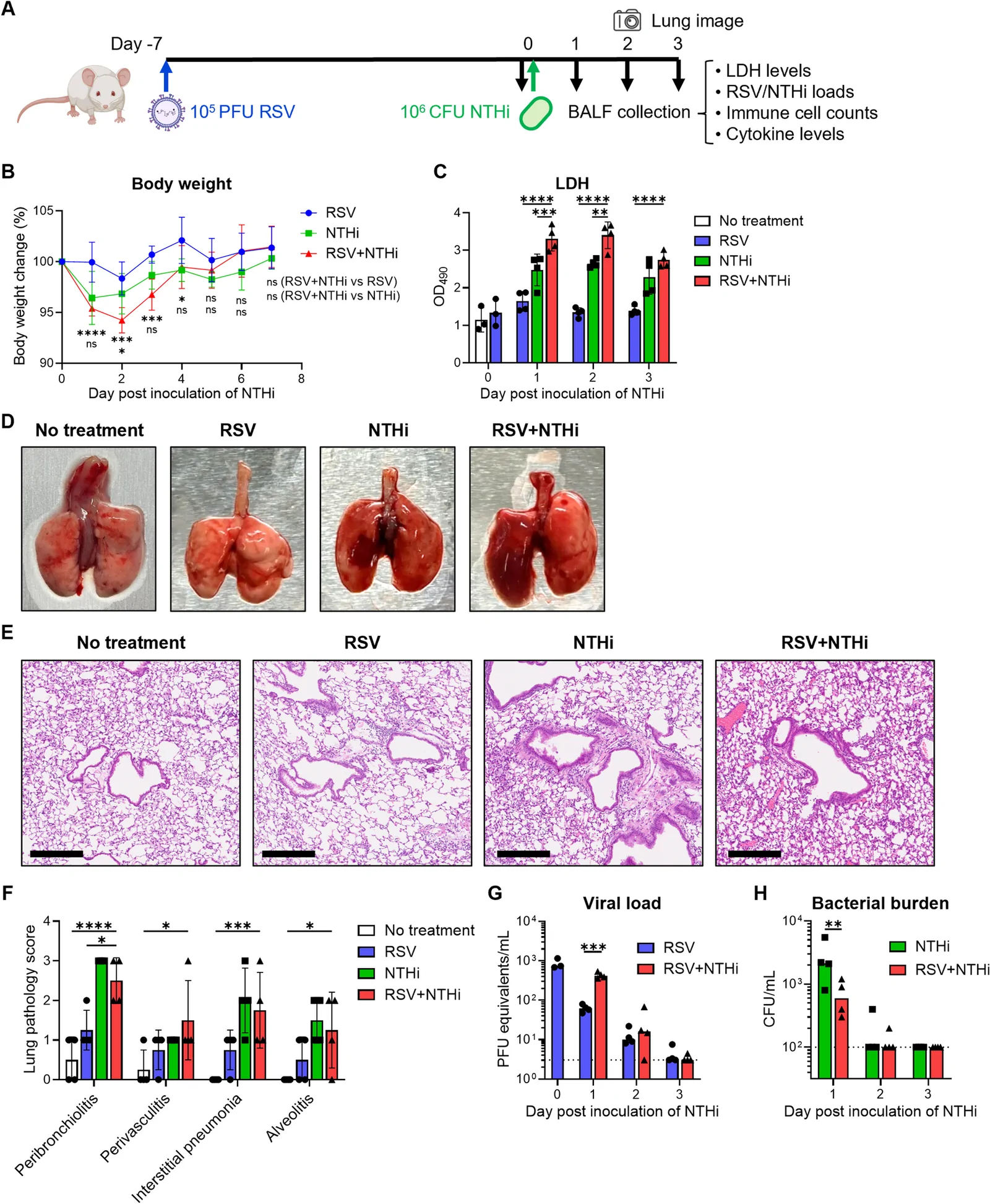
Severe lower airway inflammation induced by NTHi inoculation following RSV infection. **A**–**G** Mice were infected with respiratory syncytial virus (RSV) on day −7 and subsequently inoculated with non-typeable Haemophilus influenzae (NTHi) on day 0. **A** Experimental schedule. **B** Body weight change. **C** Levels of lactate dehydrogenase (LDH) in bronchoalveolar lavage fluid (BALF) on the indicated days. **D** Representative gross appearance of lungs on day 2 after NTHi inoculation. **E** Representative photomicrographs of lung sections stained with hematoxylin and eosin (H&E) on day 2 after NTHi inoculation. Scale bar, 250 μm. **F** Lung pathology score. **G** Viral loads in BALF on the indicated days. **H** Bacterial burden in BALF on the indicated days. **B**–**H** Each experiment was performed twice. **B** *n* = 5 per group. **C**, **G** *n* = 3–4 per group. **F**, **H** *n* = 4 per group. **B**, **C**, **F**–**H** Data are presented as (**B**, **C**, **F**) the mean ± standard deviation (SD) or (**G**, **H**) median. **B**, **C**, **F**–**H** ^*^P < 0.05, ^**^P < 0.01, ^***^P < 0.001, ^****^P < 0.0001 as indicated using two-way analysis of variance (ANOVA) with Sidak’s multiple comparison test compared with RSV+NTHi and (**C**) an unpaired two-tailed Welch’s *t* test for comparisons between the no treatment and RSV groups on day 0. **B**, **C**, **F**–**H** ns, not statistically significant. **G**, **H** Dotted lines indicate the limit of detection. For values below the limit of detection, the detection limit value was used for plotting and statistical analysis

We investigated the effect of intratracheal inoculation of NTHi on host responses following RSV infection by intranasally infecting the mice with 10^5^ PFU of RSV on day −7, followed by intratracheal inoculation with 10^6^ CFU of live NTHi (hereafter referred to as NTHi) on day 0 (Fig. 1A). The mice that received RSV followed by NTHi exhibited significant body-weight loss compared with that of mice infected with RSV alone or NTHi alone (Fig. 1B). The lowest body weight was observed on 2 days post NTHi inoculation, followed by gradual recovery (Fig. 1B).

We assessed the extent of lung injury by measuring the LDH level, which is a marker of tissue damage, in the BALF before and after NTHi inoculation (Fig. 1C). The LDH levels were significantly elevated in the RSV+NTHi group compared with that in either single-infection group on 1 and 2 days post NTHi inoculation, which indicated a more severe lung injury in the mice inoculated with NTHi following RSV infection than in the other mice (Fig. 1C). Additionally, gross examination of the lungs on 2 days post NTHi inoculation showed no apparent lesions in the RSV alone group compared with that in the untreated control group (Fig. 1D). In the NTHi alone group, patchy dark-red areas were observed on the lung surface (Fig. 1D), whereas the lungs of the mice inoculated with NTHi following RSV infection exhibited widespread dark-red areas, which indicated more extensive gross pathological involvement than that in the other mice (Fig. 1D). Moreover, histopathological analysis showed mild peribronchiolar cellular infiltration in the RSV alone group, but the extent of this change was limited compared with that observed in the untreated group (Fig. 1E, F). In contrast, the mice inoculated with NTHi following RSV infection exhibited cellular infiltration not only around the bronchioles but also in the interstitium and alveolar spaces (Fig. 1E, F). These pathological findings were more severe than those observed in the RSV alone group but were generally comparable to those observed in the NTHi alone group (Fig. 1E, F).

We evaluated pathogen dynamics in the airway by quantifying the RSV loads in the BALF using real-time qPCR and assessing the NTHi counts by plating the BALF samples. In the RSV+NTHi group, RSV level was significantly higher on 1 day post NTHi inoculation than that in the RSV alone group; however, the value was comparable to that observed on day 0 in the RSV alone group. By 2 days post NTHi inoculation, the viral loads were similar between the two groups (Fig. 1G). In contrast, the NTHi load was significantly lower in the RSV+NTHi group at 1 day post NTHi inoculation compared with that in the NTHi alone group, and NTHi was nearly undetectable in both groups by 2 days post NTHi inoculation (Fig. 1H). Collectively, these findings indicate that intratracheal inoculation with NTHi following RSV infection induced severe lower airway inflammation, which was characterized by pronounced body-weight loss, elevated LDH levels, inflammatory cell infiltration, and lung tissue injury and accompanied by sustained high RSV levels despite the reduced NTHi levels in the BALF. These results indicated successful establishment of a mouse model that exhibited severe lower airway inflammation via NTHi inoculation followed by RSV infection.

### NTHi inoculation following RSV infection induces a heightened inflammatory cytokine response in the lungs

**Fig. 2:**
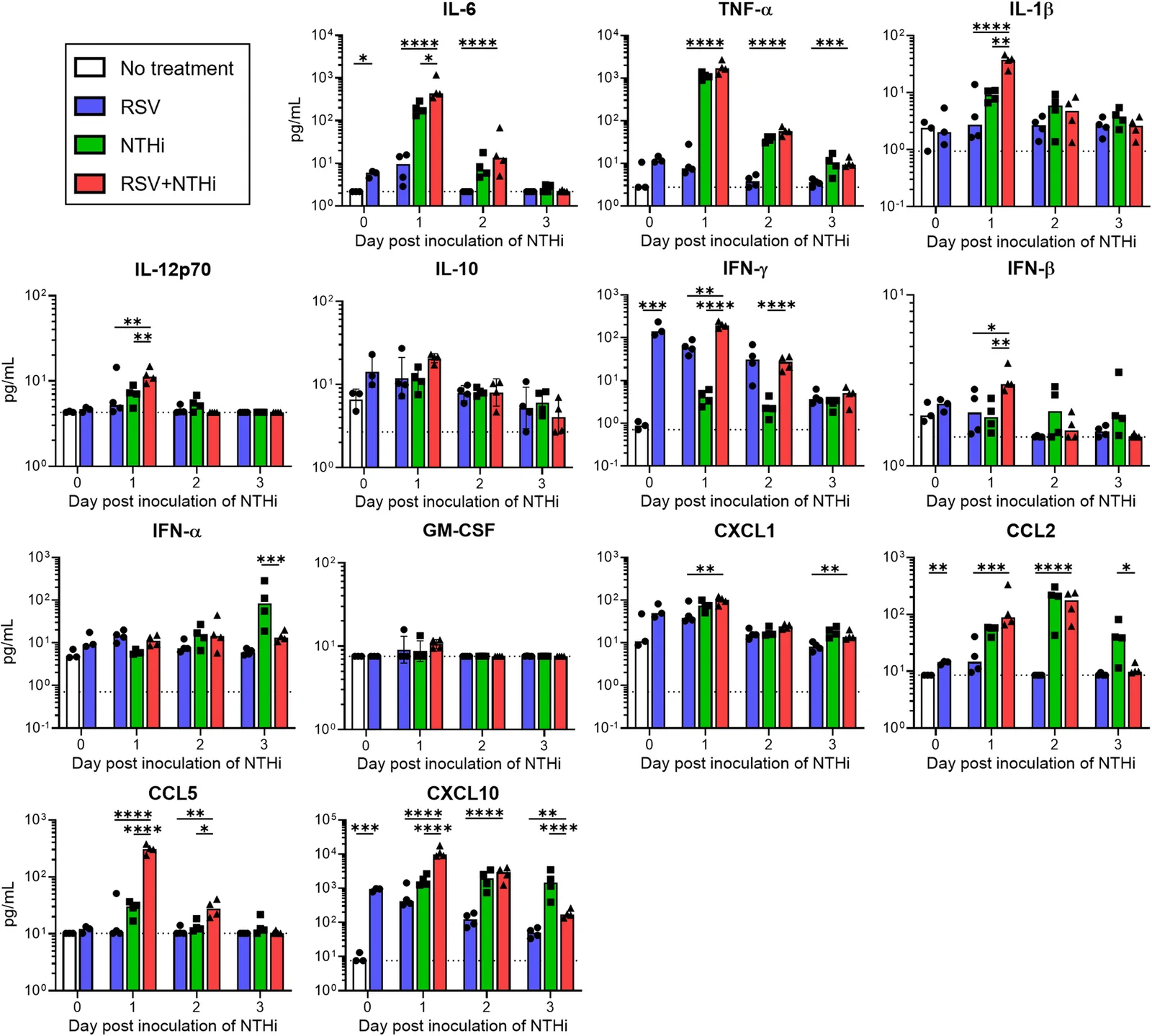
Inflammatory cytokine and chemokine responses induced by NTHi inoculation following RSV infection. Levels of interleukin (IL)-6, tumor necrosis factor (TNF)-α, IL-1β, IL-12p70, IL-10, interferon (IFN)-γ, IFN-β, IFN-α, granulocyte-macrophage colony-stimulating factor (GM-CSF), C–X–C motif chemokine ligand 1 (CXCL1), C–C motif chemokine ligand 2 (CCL2), CCL5, and CXCL10 in BALF on the indicated days after NTHi inoculation in RSV-infected mice. Each experiment was performed twice. *n* = 3–4 per group. Data are presented as the median. ^*^P < 0.05, ^**^P < 0.01, ^***^P < 0.001, ^****^P < 0.0001 as indicated using two-way ANOVA with Sidak’s multiple comparison test compared with RSV+NTHi and an unpaired two-tailed Welch’s *t* test for comparisons between the no treatment and RSV groups on day 0. Dotted lines indicate the limit of detection. For values below the limit of detection, the detection limit value was used for plotting and statistical analysis

We clarified the inflammatory profile induced by NTHi inoculation after RSV infection by measuring the inflammatory cytokine levels in the BALF before and after NTHi inoculation. At day 0, the RSV alone group exhibited significantly higher IL-6, IFN-γ, CCL2, and CXCL10 levels compared with those in the no-treatment group, which gradually declined over time (Fig. 2). At 1 day post NTHi inoculation, the RSV+NTHi group showed significantly higher IL-6, IL-1β, IL-12p70, IFN-γ, IFN-β, CXCL1, CCL5, and CXCL10 levels compared with those in both the RSV alone and NTHi alone groups (Fig. 2). The IFN-γ level in the RSV+NTHi group at this time point was comparable to that observed in the RSV alone group on day 0 (Fig. 2). Although these cytokine and chemokine levels generally declined over time, the CCL5 level at day 2 remained significantly higher than those in both the single-infection groups, whereas the IL-6, IFN-γ, and CXCL10 levels remained significantly higher than those in one of the single-infection groups (Fig. 2). TNF-α and CCL2 levels were significantly higher in the RSV+NTHi group than that in the RSV alone group on 1 and 2 days post NTHi inoculation; however, they were comparable to those in the NTHi alone group (Fig. 2). The TNF-α level remained significantly higher than that in the RSV alone group on day 3 (Fig. 2). The CXCL1 level was significantly higher than those in the RSV alone group at 1 and 3 days post NTHi inoculation (Fig. 2). The IFN-α, IL-10, and GM-CSF levels in the RSV+NTHi group did not show any detectable significant increases compared with those of both the single infection groups at any time point (Fig. 2). Taken together, these findings show that NTHi inoculation following RSV infection triggered robust pulmonary inflammatory cytokine responses.

### NTHi inoculation following RSV infection increases pulmonary CD8^+^ T-cell abundance

**Fig. 3:**
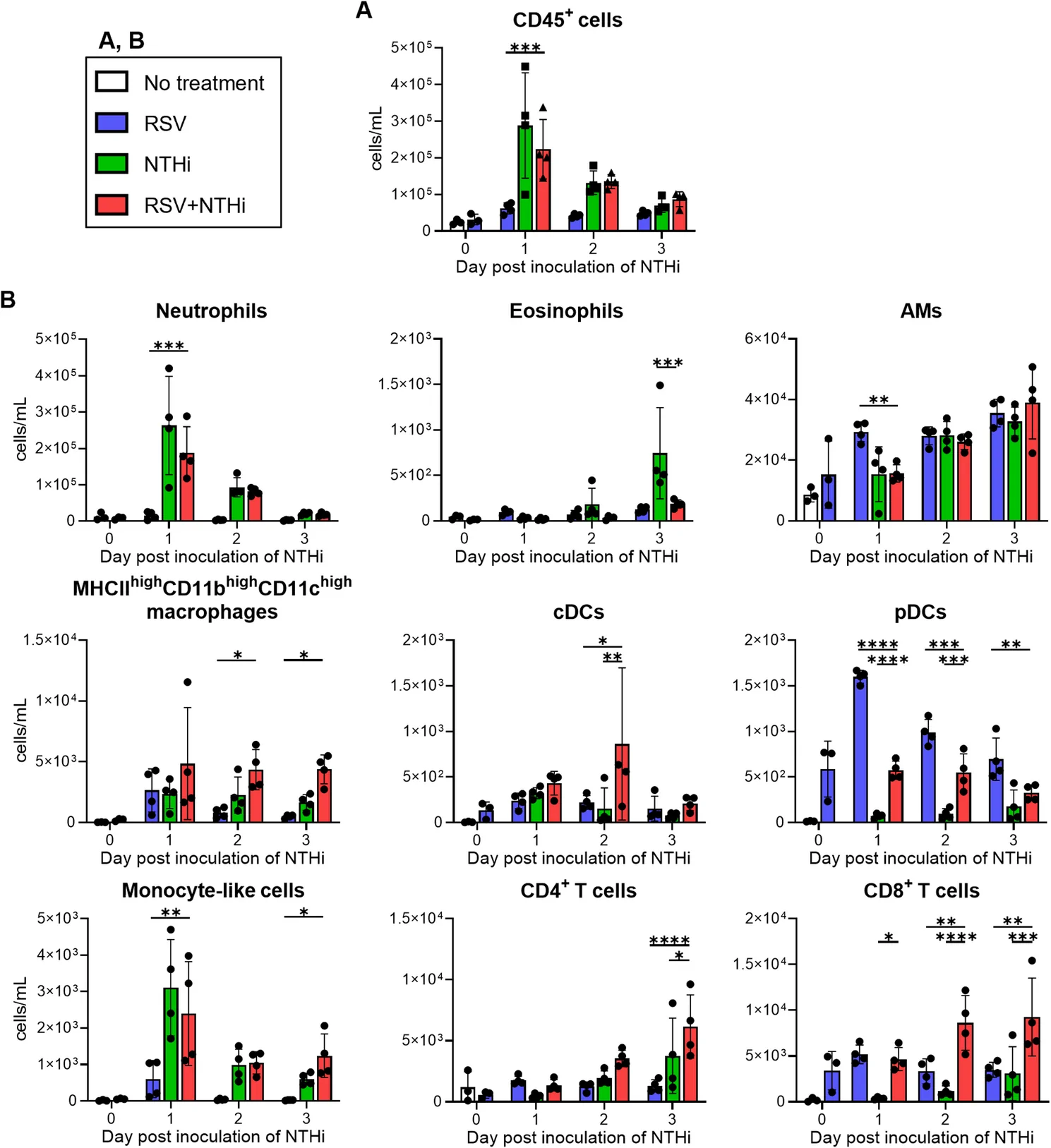
Immune-cell accumulation in BALF induced by NTHi inoculation following RSV infection. **A**, **B** Numbers of (**A**) CD45^+^ cells, (**B**) neutrophils, eosinophils, alveolar macrophages (AMs), MHCII^high^CD11b^high^CD11c^high^ macrophages, conventional dendritic cells (cDCs), plasmacytoid DCs (pDCs), CD90^−^MHCII^−^SiglecF^−^Ly6G^−^CD11b^high^ cells (monocyte-like cells), CD4^+^ T cells, and CD8^+^ T cells in BALF on the indicated days after NTHi inoculation in RSV-infected mice. Each experiment was performed twice. *n* = 3–4 per group. Data are presented as the mean ± SD. ^*^P < 0.05, ^**^P < 0.01, ^***^P < 0.001, ^****^P < 0.0001 as indicated using two-way ANOVA with Sidak’s multiple comparison test compared with RSV+NTHi and an unpaired two-tailed Welch’s t test for comparisons between the no treatment and RSV groups on day 0

We analyzed the immune cell populations in the BALF using flow cytometry (Supplementary Fig. S2, S3). The number of CD45^+^ leukocytes in the RSV+NTHi group was significantly higher than that in the RSV alone group on 1 day post NTHi inoculation but remained comparable to that in the NTHi alone group, although it subsequently declined over time (Fig. 3A). Among innate immune cells, the numbers of neutrophils, alveolar macrophages (AMs), MHCII^high^CD11b^high^CD11c^high^ macrophages, and CD90^−^MHCII^−^SiglecF^−^Ly6G^−^CD11b^high^ cells (monocyte-like cells) in the RSV+NTHi group followed patterns that were similar to those observed in the NTHi alone group, although they showed significant differences relative to those in the RSV alone group (Fig. 3B). The number of eosinophils in the RSV+NTHi group did not show any detectable significant increase compared with that in either single infection group at any time point (Fig. 3B). The number of MHCII^high^CD11b^−^CD11c^int^ cells (plasmacytoid dendritic cells, pDCs) in the RSV+NTHi group at 1 and 2 days post NTHi inoculation was significantly higher than that in the NTHi alone group but significantly lower than that in the RSV alone group (Fig. 3B). The number of MHCII^high^CD11b^int^CD11c^high^ cells (conventional dendritic cells, cDCs) reached approximately 9 × 10^2^ cells/mL and was the only innate immune subset that showed significantly higher numbers in the RSV+NTHi group at 2 days post NTHi inoculation compared with those of both single-infection groups (Fig. 3B). Among T-cell populations, the number of CD4^+^ T cells in the RSV+NTHi group was higher than those in both single-infection groups at 3 days post NTHi inoculation (Fig. 3B). The number of CD8^+^ T cells in the RSV+NTHi group was significantly higher than that in the NTHi alone group at 1 day post NTHi inoculation and comparable to that in the RSV alone group at the same time point (Fig. 3B). At 2 and 3 days post NTHi inoculation, the CD8^+^ T-cell number reached approximately 9 × 10^3^ cells/mL in the RSV+NTHi group, which was significantly higher than those in both single infection groups and substantially exceeded the number of cDCs on day 2 (Fig. 3B). These results suggest that NTHi inoculation after RSV infection preferentially increased the number of pulmonary CD8^+^ T cells, especially on day 2 post NTHi inoculation when maximal body weight loss was observed, although the total leukocyte numbers were comparable to those in the NTHi alone group.

### CD8^+^ T cells partially contribute to disease progression following NTHi inoculation in RSV-infected mice

**Fig. 4:**
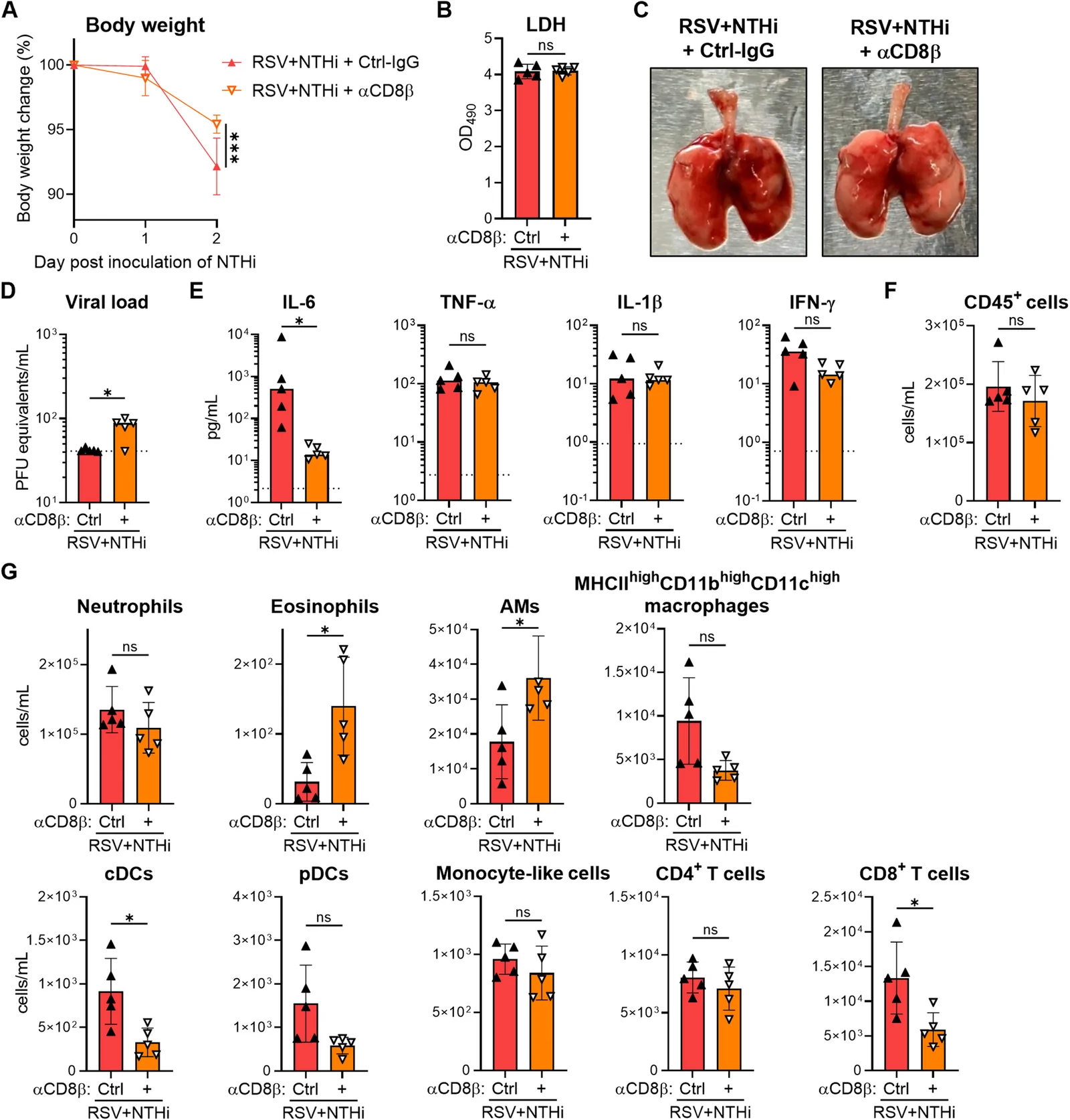
Contribution of CD8^+^ T cells to development of severe lower airway inflammation. RSV-infected mice were inoculated with NTHi and analyzed on day 2 post NTHi inoculation. Prior to NTHi inoculation, mice were treated with anti-CD8β antibody (αCD8β) or IgG1 isotype control (Ctrl-IgG). **A** Change in body weight. **B** LDH levels in BALF. **C** Representative gross appearance of lungs. **D** Viral loads in BALF. E IL-6, TNF-α, IL-1β, and IFN-γ levels in BALF. **F**, **G** Numbers of (**F**) CD45^+^ cells, (**G**) neutrophils, eosinophils, AMs, MHCII^high^CD11b^high^CD11c^high^ macrophages, cDCs, pDCs, monocyte-like cells, CD4^+^ T cells, and CD8^+^ T cells in BALF. **A**–**G** Each experiment was performed twice. **A**, **B**, **D**–**G** *n* = 5 per group. **A**, **B**, **D**–**G** Data are presented as (**A**, **B**, **F**, **G**) the mean ± SD or (**D**, **E**) median. **A**, **B**, **D**–**G** ^*^P < 0.05, ^***^P < 0.001 as indicated using (**A**) two-way ANOVA with Sidak’s multiple comparison test or (**B**, **D**–**G**) an unpaired two-tailed Welch’s *t* test. **A**, **B**, **D**–**G** ns, not statistically significant. **D**, **E** Dotted lines indicate the limit of detection. For values below the limit of detection, the detection limit value was used for plotting and statistical analysis

We assessed the contribution of CD8^+^ T cells to severe lower airway inflammation induced by NTHi inoculation after RSV infection by depleting the CD8^+^ T cells by administering an anti-CD8β antibody one day before NTHi inoculation. CD8^+^ T-cell depletion significantly improved body weight loss at 2 days post NTHi inoculation, although body weight was not fully restored (Fig. 4A). Meanwhile, in the mice inoculated with NTHi alone, CD8^+^ T-cell depletion exerted no significant effect on body weight changes (Supplementary Fig. S4). In RSV-infected mice that were subsequently inoculated with NTHi on 2 days post NTHi inoculation, CD8^+^ T-cell depletion did not significantly alter the LDH levels in the BALF (Fig. 4B); however, gross examination of the lungs showed a pronounced reduction in the dark-red lesions in CD8^+^ T cell-depleted mice compared with that observed in the isotype-treated controls (Fig. 4C). RSV levels in the BALF were significantly higher in the CD8^+^ T cell-depleted mice (Fig. 4D), whereas NTHi levels remained below the detection limit in both groups (data not shown). Additionally, the BALF IL-6 levels were significantly reduced after CD8^+^ T-cell depletion, whereas no significant changes were observed in the other measured cytokine or chemokine levels (Fig. 4E and Supplementary Fig. S5A). Flow cytometric analysis of the BALF leukocytes showed that the total CD45^+^ cell numbers were similar between the groups, and neutrophil, monocyte-like cell, and CD4^+^ T cell numbers were similarly comparable (Fig. 4F and G, and Supplementary Fig. S5B). In contrast, cDC and CD8^+^ T cell numbers were significantly reduced after CD8^+^ T-cell depletion (Fig. 4G and Supplementary Fig. S5B). MHCII^high^CD11b^high^CD11c^high^ macrophages and pDC numbers showed a decreasing trend, although the differences were not statistically significant (Fig. 4G and Supplementary Fig. S5B). Eosinophil and AM numbers were significantly increased in CD8^+^ T cell-depleted mice (Fig. 4G and Supplementary Fig. S5B). Thus, CD8⁺ T cells were considered to partially contribute to disease progression following NTHi inoculation in RSV-infected mice because CD8⁺ T-cell depletion reduced gross lung lesions, innate immune cell infiltration, and IL-6 levels but did not fully restore body weight loss or significantly reduce the BALF LDH levels.

### Live NTHi and not non-viable NTHi induces severe lower airway inflammation following RSV infection

The NTHi level in the RSV+NTHi group was significantly lower than that in the NTHi alone group and declined over time (Fig. 1H). Notably, at day 2 post NTHi inoculation, the NTHi levels were below the limit of detection in most mice (Fig. 1H), and it corresponded to peak body weight loss (Fig. 1B). Based on these observations, we performed additional analyses to examine whether bacterial viability was required for the development of severe lower airway inflammation. Specifically, the RSV-infected mice were inoculated with live NTHi, UV-killed NTHi, or heat-killed NTHi and analyzed 2 days later. At day 2 post NTHi inoculation, the RSV+live NTHi group exhibited significantly greater body weight loss compared with that of the RSV alone group, whereas the RSV + UV-killed NTHi and RSV+heat-killed NTHi groups showed body weight changes that were comparable to those observed in the RSV alone group (Supplementary Fig. S6A). At the same time point, the LDH levels in the BALF were significantly higher in the RSV + UV-killed NTHi and RSV+heat-killed NTHi groups than that in the RSV-alone group but remained significantly lower than that in the RSV+live NTHi group (Supplementary Fig. S6B). The BALF RSV levels did not differ significantly among the groups; however, the RSV + UV-killed NTHi group showed a modest increasing trend compared with that of the RSV alone group (Supplementary Fig. S6C). Analysis of cytokines and chemokines in BALF showed that mice inoculated with UV- or heat-killed NTHi exhibited overall weaker inflammatory responses than those inoculated with live NTHi, with live NTHi inducing a more pronounced IL-6-dominant inflammatory cytokine response (Supplementary Fig. S6D, S7). A similar trend was observed for chemokine responses, with stronger inflammatory signaling detected in the RSV+live NTHi group than in the other groups (Supplementary Fig. S7). Flow cytometric analysis of the BALF leukocytes showed that the number of CD45^+^ cells in RSV-infected mice inoculated with UV- or heat-killed NTHi was significantly higher than that in the RSV alone group but significantly lower than that in the RSV+live NTHi group (Supplementary Fig. S6E, S8). The analysis of leukocyte subsets showed that neutrophil, MHCII^high^CD11b^high^CD11c^high^ macrophage, monocyte-like cell, and T cell numbers were significantly increased in at least one of the RSV + UV-killed NTHi and RSV+heat-killed NTHi groups compared with the RSV-alone group (Supplementary Fig. S6F, S8). However, neutrophil accumulation in the RSV + UV-killed NTHi and RSV+heat-killed NTHi groups was substantially lower than that in the RSV+live NTHi group, whereas MHCII^high^CD11b^high^CD11c^high^ macrophage, monocyte-like cell, and T cell numbers were comparable to those in the RSV+live NTHi group (Supplementary Fig. S6F, S8). Taken together, these results indicated that live NTHi induced stronger lower airway inflammation characterized by enhanced inflammatory mediator production and neutrophil recruitment following RSV infection than that observed for non-viable NTHi.

Despite this, inoculation with UV- or heat-killed NTHi led to a comparable increase in the pulmonary CD8^+^ T cell numbers to those observed in the RSV+live NTHi group (Supplementary Fig. S6F, S8); however, these mice did not exhibit body weight loss (Supplementary Fig. S6A). These findings suggested that CD8^+^ T-cell accumulation alone was insufficient to explain disease exacerbation. Therefore, we further examined whether live and non-viable NTHi differentially affected the functional phenotype of CD8^+^ T cells. To address this question, we analyzed cytokine production by CD11a^high^CD44^high^CD8^+^ T cells using flow cytometry in RSV-infected mice 2 days after inoculation with live or UV-killed NTHi (Supplementary Fig. S9). CD11a is a well-established marker of antigen-experienced and activated CD8^+^ T cells [31–33]. Compared with that of the RSV alone group, the RSV+live NTHi group showed significantly increased frequencies of IFN-γ-, TNF-α-, or granzyme B-producing CD8^+^ T cells (Supplementary Fig. S10A, S10B). In contrast, the RSV + UV-killed NTHi group did not show a significant increase in the number of CD8^+^ T cells producing IFN-γ, granzyme B, or TNF-α. Furthermore, the population of multifunctional CD8^+^ T-cell subsets, including IFN-γ^+^TNF-α^+^ and IFN-γ^+^granzyme B^+^ populations, did not significantly increase in either the RSV+live NTHi or RSV + UV-killed NTHi groups compared with that in the RSV alone group (Supplementary Fig. S10A, S10B). Taken together, these findings suggested that although CD8^+^ T cells contributed to the development of severe lower airway inflammation, their accumulation alone was insufficient to drive disease severity. Instead, additional inflammatory factors induced by live NTHi likely played a critical role in the development of severe pathology.

### Pulmonary IL-6 levels correlate with disease severity across distinct RSV–NTHi conditions

**Fig. 5:**
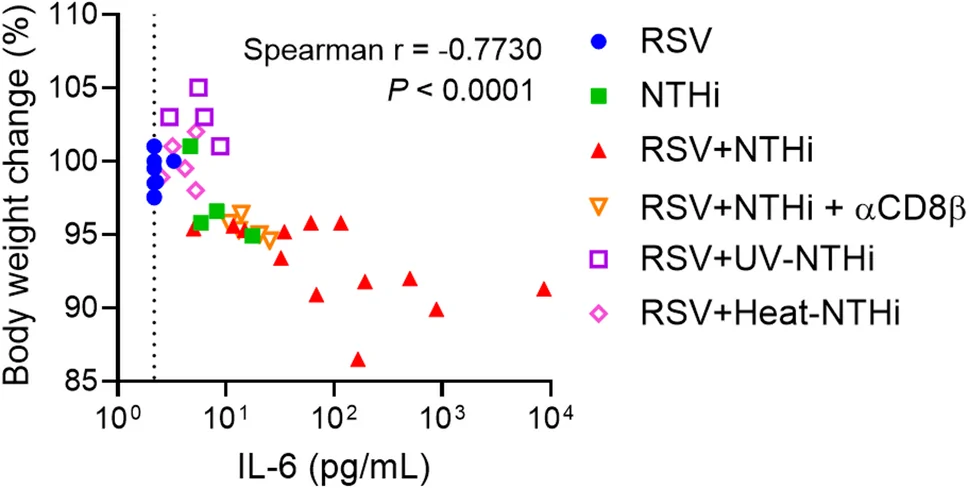
Correlation between body weight loss and BALF IL-6 levels. Scatter plot showing the correlation between body weight loss and IL-6 levels in BALF on day 2 post NTHi inoculation in RSV-infected mice. Each dot represents an individual mouse and is color-coded according to the indicated experimental group. Correlation was assessed using Spearman’s rank correlation test using all data points. Dotted lines indicate the limit of detection. For values below the limit of detection, the detection limit value was used for plotting and statistical analysis

Based on the observation that CD8^+^ T-cell accumulation alone was insufficient to explain disease severity, we examined whether inflammatory mediators better reflected the clinical outcomes. As the IL-6 level consistently differed among the experimental groups, we focused on the relationship between pulmonary IL-6 level and body weight loss. We integrated IL-6 concentrations in the BALF and body weight changes from all experimental conditions examined in this study such as RSV alone, NTHi alone, RSV+live NTHi, RSV + UV-killed NTHi, RSV+heat-killed NTHi, and RSV+live NTHi after CD8^+^ T-cell depletion. We plotted change in body weight (percentage of baseline) against IL-6 concentration and observed a strong negative correlation (Spearman *r* = -0.7730, *P* < 0.0001; Fig. 5). Higher IL-6 levels were consistently associated with greater body weight loss, whereas conditions with lower IL-6 levels showed minimal changes in body weight (Fig. 5). These findings showed that pulmonary IL-6 level closely reflected disease severity across distinct experimental settings. Additionally, they support IL-6 as a potential biomarker associated with inflammatory severity in RSV-NTHi-associated lower airway inflammation.

## Discussion

In this study, we have shown that NTHi inoculation after RSV infection induces severe lower airway inflammation in a murine model. NTHi alone strongly recruits neutrophils and monocyte-like cells and induces inflammatory cytokines such as IL-6 and TNF-α, which is consistent with previously reported findings [34–36]. NTHi inoculation after RSV infection resulted in lower NTHi levels in the BALF than that observed for inoculation with NTHi alone, whereas innate immune responses characteristic of NTHi infection alone were reproduced along with sustained high RSV levels, pronounced CD8^+^ T-cell accumulation, and further amplification of inflammatory mediators centered on IL-6. This pattern of changes in microbial levels in the BALF contrasts with classical RSV-pneumococcal models, which are typically characterized by enhanced pneumococcal colonization and proliferation following RSV infection [1, 37, 38]. Furthermore, we have shown that CD8⁺ T cells partially contribute to disease progression while additionally participating in RSV clearance: their depletion reduces gross lung lesions, innate immune cell infiltration, and IL-6 levels but does not fully restore body weight loss or significantly reduce the BALF LDH levels. Additionally, UV- or heat-killed NTHi induced CD8^+^ T-cell accumulation to comparable levels to those induced by live NTHi; however, they do not induce body weight loss or robust production of inflammatory mediators. Moreover, the BALF IL-6 levels show a strong correlation with body weight loss, which suggests that IL-6 is associated with disease severity. Collectively, these findings suggest that live NTHi inoculation following RSV infection exacerbates lower airway inflammation through combined inflammatory responses involving sustained high RSV levels, innate immune cell recruitment, CD8⁺ T-cell accumulation, and increased levels of inflammatory mediators such as IL-6.

We found that NTHi inoculation following RSV infection resulted in sustained elevated RSV levels at 1 day post-NTHi inoculation compared with that observed after RSV infection alone. Although previous studies have primarily focused on the manner in which RSV infection promotes bacterial adhesion or colonization [18–23], the effect of secondary NTHi inoculation on RSV replication has not been well characterized. Clinically, increased RSV viral loads have been reported in patients with severe RSV with concomitant detection of *H. influenzae* [9, 10], and our results are consistent with these clinical observations. The tendency toward increase in RSV levels was observed even in the RSV + UV-killed NTHi group, which suggests that bacterial structural components may contribute to modulation of the RSV growth environment. Additionally, reduced pDC and AM numbers in the RSV+live NTHi group may have contributed to sustained RSV levels owing to their roles in antiviral immunity and RSV clearance [39, 40]. In contrast, prior RSV infection promoted a decrease in NTHi levels in the BALF in our model, which differed from that observed in influenza virus models, where secondary NTHi infection enhances bacterial proliferation and persistence in the lung [41, 42]. Previous in vitro studies have reported that RSV infection enhances NTHi adhesion to airway epithelial cells [18–23]. However, these studies primarily focused on epithelial adhesion and did not sufficiently evaluate bacterial survival or immune cell-mediated clearance within the airway lumen. Therefore, differences in experimental systems and outcome measures—particularly the absence of immune cell-dependent clearance mechanisms in vitro—may have contributed to the discrepancy between previous reports and our findings. Moreover, our analyses were based on BALF samples; hence, the NTHi adherent to epithelial surfaces or residing within lung tissue may not have been fully recovered. Therefore, future studies are needed to directly evaluate epithelial attachment and tissue localization of NTHi during RSV-associated lower airway inflammation.

NTHi inoculation following RSV infection was accompanied by a pronounced increase in pulmonary CD8^+^ T cell levels. Based on our findings, this accumulation may have been associated with elevated CCL5 and CXCL10 levels [43–45]. Additionally, sustained high RSV levels may enhance antigen presentation by infected epithelial and antigen-presenting cells, thereby promoting antigen-dependent activation and expansion of RSV-specific CD8^+^ T cells. Furthermore, we have shown that CD8^+^ T cells partially contribute to the exacerbation of lower airway inflammation in this model. Previous studies have reported that RSV-specific CD8^+^ T cells promote body weight loss and aggravate pulmonary pathology after RSV challenge, with IFN-γ playing a central role in mediating these pathological effects [32]. In our model, IFN-γ levels were higher in the RSV+NTHi group than that in the RSV-alone group at day 1 post NTHi inoculation. However, CD8^+^ T-cell depletion did not significantly alter overall IFN-γ levels, which may be reflective of compensatory contributions from other IFN-γ-producing populations such as CD4^+^ T cells and natural killer cells. In contrast, CD8^+^ T-cell depletion significantly reduced the pulmonary IL-6 levels. IL-6 is released from epithelial cells, macrophages, and other stromal cells in response to tissue injury and inflammatory stimuli [1, 46]. Therefore, IL-6 production in this model may be amplified secondary to CD8^+^ T cell-mediated cytotoxic destruction of infected cells, leading to further propagation of inflammatory signaling. This CD8^+^ T cell–IL-6 axis may be an important amplification loop that links antiviral immune responses to excessive pulmonary inflammation. Therefore, we suggest that CD8^+^ T cells contribute to the induction of an inflammatory pulmonary environment after NTHi inoculation in RSV-infected mice, potentially through IL-6-associated inflammatory amplification.

NTHi outer membrane components such as the lipoprotein P6 and lipooligosaccharide (LOS) activate Toll-like receptor 2 and 4 signaling in airway epithelial cells and alveolar macrophages [34, 47, 48]. Activation of these pathways promotes neutrophil recruitment and induces the production of proinflammatory cytokines such as TNF-α [14, 34, 48, 49]. In concurrence with these findings, UV- and heat-killed NTHi retained the ability to induce inflammatory cytokines, chemokines, and immune cell recruitment. However, after RSV infection, only live NTHi and not UV- or heat-killed NTHi induced body weight loss and robust production of inflammatory mediators. This observation suggests that it is bacterial viability, rather than outer membrane components alone, that is associated with the development of severe lower airway inflammation. Although secreted bacterial products (such as outer membrane vesicles (OMVs)) or intracellular invasion may be involved, these mechanisms remain speculative and require further investigation.

Our data showed that CD8^+^ T-cell depletion resulted in only slight improvement in body weight and did not significantly reduce the BALF LDH levels. Additionally, under conditions of prior RSV infection, UV-killed NTHi or heat-killed NTHi induced CD8^+^ T-cell accumulation to a comparable level to that induced by live NTHi, although body weight loss was not observed. These results suggest that CD8^+^ T-cell accumulation alone is insufficient to cause severe pathology and that the involvement of other inflammatory factors is required for disease progression. In fact, under conditions of prior RSV infection, live NTHi induced stronger neutrophil infiltration than that induced by non-viable NTHi, which suggests that neutrophilic inflammation may additionally contribute to disease pathogenesis. Therefore, this model indicates that CD8^+^ T cells partially contribute to disease progression; however, other inflammatory pathways including those associated with innate immune responses are additionally likely involved in severe pathology.

Pulmonary IL-6 levels showed a strong correlation with the degree of body weight loss, which suggests that IL-6 is associated with disease severity. These findings are consistent with clinical observations, where co-detection of *H. influenzae* in patients with severe RSV infection was associated with elevated IL-6 levels [10]. Additionally, elevated IL-6 levels in circulation or the lungs have been associated with disease severity and clinical outcomes in viral infections such as COVID-19 [50–52]. However, our data are correlative; hence, future studies using IL-6- or IL-6 receptor-targeted interventions are warranted to determine whether IL-6 contributes to disease exacerbation or reflects ongoing inflammatory responses.

Some limitations of this study should be considered when interpreting its translational relevance. First, the airway microbiota differs substantially in both composition and ecological structure between mice and humans, which may influence host–pathogen and host–microbiota interactions during RSV infection. Additionally, we cannot exclude the possibility that resident microorganisms other than NTHi or changes in their composition or function may have contributed to the inflammatory responses observed in this model. Therefore, further studies using gnotobiotic or microbiota-controlled models are needed to clarify the contribution of the endogenous microbiota to RSV–NTHi interactions. Second, this study only used 6-week-old female BALB/c mice, which may limit the generalizability of our findings. However, RSV infection studies widely use young adult female BALB/c mice that are typically 6–8 weeks of age; hence, using this standardized model helps minimize biological variability. However, these findings must be validated in future studies using mice of both sexes, different age groups, and other strains. Third, our model does not fully recapitulate the natural route of entry of NTHi to the lower respiratory tract in humans. NTHi is a commensal bacterium of the upper airway in children and adults [14, 53], and its entry into the lower airway may occur through aspiration or through bacterial overgrowth and subsequent spread associated with alterations in the upper-airway microbiota. In this study, we artificially introduced the bacteria into the lower airway by directly administering NTHi intratracheally as a bolus inoculum. Despite these limitations, this model provides an experimental framework for directly evaluating the inflammatory consequences of bacterial entry into the lung during RSV infection and reinterpreting clinical observations of RSV–*H. influenzae* co-detection.

## Conclusions

This study shows that live NTHi inoculation following RSV infection induces severe lower airway inflammation in a murine model. This pathology showed association with sustained high RSV levels, innate immune cell recruitment, CD8^+^ T-cell accumulation, and increased IL-6 levels. We have inferred that CD8^+^ T cells partially contribute to disease progression and that IL-6 is a potential biomarker associated with disease severity. Overall, these findings suggest a novel pathological role for NTHi in the exacerbation of RSV-associated lower airway inflammation.

## Supplementary Information

Supplementary Material 1.

Supplementary Material 2.

## Data Availability

The datasets used and/or analyzed during the current study are available from the corresponding author upon reasonable request.

## Abbreviations

AM: Alveolar macrophage
ANOVA: Analysis of variance
BALF: Bronchoalveolar lavage fluid
β-NAD: β-nicotinamide adenine dinucleotide
CCL: C–C motif chemokine ligand
cDC: Conventional dendritic cell
cDNA: Complementary DNA
CFU: Colony-forming unit
CXCL: C–X–C motif chemokine ligand
DMSO: Dimethyl sulfoxide
FBS: Fetal bovine serum
GM-CSF: Granulocyte–macrophage colony-stimulating factor
H&E: Hematoxylin and eosin
IFN: Interferon
IL: Interleukin
LDH: Lactate dehydrogenase
LOS: Lipooligosaccharide
NGS: Next-generation sequencing
NTHi: Non-typeable Haemophilus influenzae (live bacteria unless otherwise specified)
OMV: Outer membrane vesicle
PBS: Phosphate-buffered saline
PCR: Polymerase chain reaction
pDC: Plasmacytoid dendritic cell
PFU: Plaque-forming unit
qPCR: Quantitative polymerase chain reaction
RSV: Respiratory syncytial virus
SD: Standard deviation
TBE: Tris borate ethylenediamine tetracetic acid
TNF: Tumor necrosis factor
UV: Ultraviolet
