# Supplementary material for: Live nontypeable Haemophilus influenzae exacerbates respiratory syncytial virus-associated lower airway inflammation
Source: Respir Res. 2026 Jun 20;27:353. doi: 10.1186/s12931-026-03774-4 (PMC13584502; doi:10.1186/s12931-026-03774-4)
Supplement: Supplementary file 1 [file 12931_2026_3774_MOESM1_ESM.docx]

# Supplementary Figures

**Supplementary Fig. S1. Identification of NTHi strain** Genomic DNA extracted from *H. influenzae* UT-16973 (clinical isolate) and *H. influenzae* serotype b (Hib) were amplified using PCR with the primers listed in Supplementary Table S1, followed by agarose gel electrophoresis. The expected amplicon sizes were 343 bp for *bexA*, 567 bp for *bexB*, and 918 bp for *pepN*. M: 100 bp DNA ladder.

**Supplementary Fig. S2. Representative gating strategy for longitudinal flow cytometry analysis of immune cells in BALF**

**Supplementary Fig. S3. Flow cytometric analysis of immune cells in BALF after NTHi inoculation following RSV infection** Representative gating plots. The numbers shown in the plots reflect the number of cells in the gate. This experiment was performed twice.

**Supplementary Fig. S4. Body weight changes after NTHi inoculation in mice with or without CD8^+^ T cell depletion** This experiment was performed twice. n = 5 per group. Data are presented as the mean ± SD. Data were analyzed using two-way ANOVA with Sidak’s multiple-comparison test. ns, not statistically significant.

**Supplementary Fig. S5. Altered immune responses following CD8^+^ T-cell depletion** RSV-infected mice were inoculated with NTHi and analyzed on day 2 post NTHi inoculation. Prior to NTHi inoculation, mice were treated with anti-CD8β antibody (αCD8β) or IgG1 isotype control (Ctrl-IgG). **A** IL-12p70, IL-10, IFN-β, IFN-α, GM-CSF, CXCL1, CCL2, CCL5, and CXCL10 levels in BALF. **B** Representative gating plots. The numbers shown in the plots reflect the number of cells in the gate. **A**, **B** Each experiment was performed twice. **A** *n* = 5 per group. Data are presented as the median. Data were analyzed using an unpaired two-tailed Welch’s t test. ns, not statistically significant. Dotted lines indicate the limit of detection. For values below the limit of detection, the detection limit value was used for plotting and statistical analysis.

**Supplementary Fig. S6. Effects of live NTHi on development of severe lower airway inflammation following RSV infection** RSV-infected mice were inoculated with live NTHi, UV-killed NTHi, or heat-killed NTHi and analyzed on day 2 post inoculation. **A** Body weight change. **B** LDH levels in BALF. **C** Viral loads in BALF. **D** IL-6, TNF-α, IL-1β, and IFN-γ levels in BALF. **E**, **F** Numbers of (**E**) CD45^+^ cells, (**F**) neutrophils, eosinophils, AMs, MHCII^high^CD11b^high^CD11c^high^ macrophages, cDCs, pDCs, monocyte-like cells, CD4^+^ T cells, and CD8^+^ T cells in BALF. **A**–**F** Each experiment was performed twice. **A** *n* = 5 per group. **B**–**F** *n* = 4–5 per group after exclusion of samples with failed BALF collection. **A**–**F** Data are presented as (**A**, **B**, **E**, **F**) the mean ± SD or (**C**, **D**) median. **A**–**F** ^*^*P* < 0.05, ^**^*P* < 0.01,^***^*P* < 0.001, ^****^*P* < 0.0001 as indicated using (**A**) two-way ANOVA with Dunnett’s multiple comparison test or (**B**–**F**) one-way ANOVA with Tukey’s multiple comparison test. **A**–**F** ns, not statistically significant. **C**, **D** Dotted lines indicate the limit of detection. For values below the limit of detection, the detection limit value was used for plotting and statistical analysis.

**Supplementary Fig. S7. IL-12p70, IL-10, IFN-β, IFN-α, GM-CSF, CXCL1, CCL2, CCL5, and CXCL10 levels in BALF on day 2 after inoculation with live, UV-killed, or heat-killed NTHi in RSV-infected mice** This experiment was performed twice. *n* = 4–5 per group after exclusion of samples with failed BALF collection. Data are presented as the median. ^*^*P* < 0.05, ^**^*P* < 0.01, ^***^*P* < 0.001, ^****^*P* < 0.0001 as indicated using one-way ANOVA with Tukey’s multiple comparison test. ns, not statistically significant. Dotted lines indicate the limit of detection. For values below the limit of detection, the detection limit value was used for plotting and statistical analysis.

**Supplementary Fig. S8. Flow cytometric analysis of immune cells in BALF after NTHi inoculation following RSV infection** Representative gating plots are shown. The numbers shown in the plots reflect the number of cells in the gate. This experiment was performed twice.

**Supplementary Fig. S9. Representative gating strategy for flow cytometric phenotypic analysis of CD8^+^ T cells in BALF following inoculation of live or UV-killed NTHi after RSV infection**

**Supplementary Fig. S10. Phenotypic analysis of CD8^+^ T cells in BALF following inoculation of live or UV-killed NTHi after RSV infection** Cytokine-producing CD11a^high^CD44^high^CD8^+^ T cells in BALF were analyzed 2 days after administration of live or UV-killed NTHi in RSV-infected mice. Brefeldin A was administered 6 h prior to sample collection. **A** Representative gating plots. The numbers shown in the plots reflect the number of cells in the gate. **B** Number of cytokine-producing CD11a^high^CD44^high^CD8^+^ T cells in BALF. **A**, **B** Each experiment was performed twice. **B** *n* = 3–4 per group; one sample was excluded because counting beads were not added during flow cytometric acquisition, preventing calculation of absolute cell numbers. Data are presented as the mean ± SD. ^*^*P* < 0.05 as indicated using one-way ANOVA with Tukey’s multiple comparison test. ns, not statistically significant.
