# Supplementary material for: Live nontypeable Haemophilus influenzae exacerbates respiratory syncytial virus-associated lower airway inflammation
Source: Respir Res. 2026 Jun 20;27:353. doi: 10.1186/s12931-026-03774-4 (PMC13584502; doi:10.1186/s12931-026-03774-4)
Supplement: Supplementary file 2 [file 12931_2026_3774_MOESM2_ESM.docx]

# Supplementary Tables

**Supplementary Table S1. Primer sequences**

**Supplementary Table S2. Antibodies used for flow cytometry**
